# Trauma, mental health, and intergenerational associations in Kosovar Families 11 years after the war

**DOI:** 10.3402/ejpt.v4i0.21060

**Published:** 2013-08-13

**Authors:** Matthis Schick, Naser Morina, Richard Klaghofer, Ulrich Schnyder, Julia Müller

**Affiliations:** Department of Psychiatry and Psychotherapy, University Hospital Zürich, Switzerland

**Keywords:** PTSD, trauma, Kosovo, war, intergenerational, children, parents

## Abstract

**Background:**

While there is a considerable amount of literature addressing consequences of trauma in veterans and holocaust survivors, war and postwar civilian populations, particularly children, are still understudied. Evidence regarding intergenerational effects of trauma in families is inconsistent.

**Objective:**

To shed light on intergenerational aspects of trauma-related mental health problems among families 11 years after the Kosovo war.

**Method:**

In a cross-sectional study, a paired sample of 51 randomly selected triplets (school-aged child, mother, father, *N*=153) of Kosovar families was investigated with regard to trauma exposure, posttraumatic stress (UCLA Posttraumatic Diagnostic Scale), anxiety (Spence Children's Anxiety Scale, Hopkins Symptom Checklist-25), and depressive symptoms (Depressionsinventar für Kinder und Jugendliche [DIKJ; depression inventory for children and adolescents], Hopkins Symptom Checklist-25).

**Results:**

Considerable trauma exposure and high prevalence rates of clinically relevant posttraumatic stress, anxiety, and depressive symptoms were found in both parents and children. While strong correlations were found between children's depressive symptoms and paternal posttraumatic stress, anxiety and depressive symptoms, maternal symptoms did not correlate with their children's. In multiple regression analyses, only posttraumatic stress symptoms of fathers were significantly related with children's depressive symptoms.

**Conclusion:**

Eleven years after the Kosovo war, the presence of posttraumatic stress, anxiety, and depressive symptoms in civilian adults and their children is still substantial. As symptoms of parents and children are associated, mental health problems of close ones should be actively screened and accounted for in comprehensive treatment plans, using a systemic approach. Future research should include longitudinal studies conducting multivariate analyses with larger sample sizes in order to investigate indicators, causal and resilience factors.

Civilian populations all over the world have become principal targets of military and paramilitary persecution and thereby victims of severely traumatizing experiences, particularly during the many ethnically motivated armed conflicts of recent years (International Committee of the Red Cross, 2010). People concerned have been reported to show an increased prevalence of mental disorders, namely posttraumatic stress disorder (PTSD), anxiety and depression (Cardozo et al., 2004; Cardozo, Vergara, Agani, & Gotway, 2000; Johnson & Thompson, 2008; Neuner et al., 2004; Schaal, Dusingizemungu, Jacob, & Elbert, 2011; Scholte et al., 2004). Notably, children were shown to frequently suffer from mood and anxiety disorders, especially PTSD, in the aftermath of war (Ahmad, Von Knorring, & Sundelin-Wahlsten, 2008; Hadi, Llabre, & Spitzer, 2006) and must be considered as particularly vulnerable. Besides traumatic experiences, the affected populations often suffer from difficult war and postwar living conditions which increase the risk of developing psychological symptoms (Heptinstall, Sethna, & Taylor, 2004; King, King, Fairbank, Keane, & Adams, 1998; Klaric, Klaric, Stevanovic, Grkovic, & Jonovska, 2007; Miller et al., 2002; Wenzel et al., 2009). Without appropriate treatment, which is often unavailable in postwar countries, the majority of individuals with chronic PTSD do not recover (Goenjian et al., 2000; Kessler, Sonnega, Bromet, Hughes, & Nelson, 1995b).

The 1998/1999 war in Kosovo represents one of the few conflict zones that were rather closely observed by western media and politics and therefore followed by comparably fast and extensive support in terms of restoration of civil order and infrastructure. Nevertheless, high short- and medium-term postwar prevalence rates of mental disorders have been observed (Kashdan, Morina, & Priebe, 2009; Morina & Ford, 2008). A large epidemiological survey conducted in the Balkans found a prevalence for mood and anxiety disorders of 47.6% and 41.8%, respectively, in 648 Kosovar adults (Priebe et al., 2010).

Moreover, not only those directly exposed to trauma but also their close ones can be affected in a broad range of ways. Children of fathers involved in the first Gulf war have been shown to present with elevated scores of anxiety and depression (Al-Turkait & Ohaeri, 2008). PTSD in Vietnam veterans predicted their offspring's behavioral problems (Caselli, 1995). Veterans with war-related PTSD significantly more often reported developmental, behavioral, and emotional problems in their children than veterans without PTSD (Klarić et al., 2008). More than a third of war veterans’ wives were found to meet the criteria for secondary traumatic stress (Francisković et al., 2007) and veterans’ PTSD was related to lower levels of marital adjustment (Klaric et al., 2011). A variety of intergenerational effects of parental trauma exposure and posttraumatic stress symptoms on parenting, child development, and family functioning in veterans and holocaust survivors have been reviewed (Dekel & Goldblatt, 2008; Galovski & Lyons, 2004; Kellerman, 2001), often with inconsistent findings.

Several studies examined intergenerational aspects in families in which both parents and children were exposed to traumatic experiences. Both war trauma and parents’ emotional responses in terms of PTSD and anxiety were found to be significantly associated with children's PTSD and anxiety symptoms in 100 traumatized families from the Gaza Strip (Thabet, Tawahina, El Sarraj, & Vostanis, 2008). In 296 Tamil school children, a significant relationship between previous war events and the amount of family violence experienced by the children was reported. Both violence associated with the war and with parental behavior were in turn related to the diagnosis of PTSD in children (Catani, Jacob, Schauer, Kohila, & Neuner, 2008). Self-reported data of 339 mothers and children from Bosnia-Herzegovina revealed high levels of posttraumatic stress symptoms in mothers and children. Child distress was related to both their level of exposure and to maternal posttraumatic stress reactions (Smith, Perrin, Yule, & Rabe-Hesketh, 2001).

While there is a considerable amount of literature addressing veterans and holocaust survivors, war and postwar civilian populations, particularly children, are still understudied. Furthermore, while earlier studies were usually conducted on the impact on children of a single traumatized parent (mostly fathers) or both parents together, few studies have examined traumatized families in terms of the differential relationship of each parent's and children's individual mental health problems. This cross-sectional study, therefore, aimed at shedding light on the intergenerational interplay of PTSD, anxiety, and depressive symptoms among Kosovar families 11 years after the war. We hypothesized that, despite the relatively long time lapse since the end of war, both children and adults would still suffer to a great extent from clinically relevant symptoms of posttraumatic stress, anxiety, and depression, with children being less affected than their parents due to lower lifetime trauma exposure. Given the inconsistent evidence regarding the influence of maternal versus paternal symptoms on their children's well-being, we expected equally strong correlations between the psychopathological symptoms of the children and their mothers’ and fathers’, respectively.

## Methods

The ethics committee of the County of Zurich, Switzerland approved the study. As no ethics committee was available in Kosovo, the study was submitted to and approved by the Ministry of Education of Kosovo. Data collection occurred from May to November 2010.

### Participants and procedure

Participants, that is, children and their parents, were recruited from the general population of three different regions of Kosovo (north, central, and south) in order to avoid bias resulting from town–country discrepancy or different degrees of involvement in war. We included Albanian families with children born during or before the war (i.e., aged 10–17 years at the time of data collection) and both parents living in the same household. Families were excluded if they had not been permanently resident in Kosovo or if higher military functions were executed during the war. School authorities of 14 communities were asked for permission to contact the principals of 17 elementary schools and nine high schools randomly selected from a list provided by the Ministry of Education. In the case of agreement, school principals and teachers were approached by the study team (N. M.) in order to obtain permission to ask students for cooperation. Students from 5th to 11th grade were randomly selected from class lists and asked for participation. In the case of consent, parents were subsequently addressed on their part. Written informed consent was obtained from all participating children and parents. Children were mostly interviewed at school, whereas parents were interviewed at their homes. Interviews were conducted by six master-level students of psychology from the University of Prishtina. They were thoroughly trained in a two-day workshop to correctly provide study information, obtain informed consent, and conduct interviews according to the study protocol. A member of the research team (N. M.) provided on-site support and supervision during the first week of data collection. Another half-day supervision session in a group setting took place six weeks later. Telephone and mail support was granted during the complete study period. Parents obtained €10 for completing the interviews, children €5. Interviewers obtained €12 for each completed interview.

All contacted school authorities, principals, and teachers granted full support without exception. From 129 students approached by the study team, 114 (88%) agreed to participate. Among these, parents of 18 children (16%) entirely refused cooperation, whereas either the mother or father of 45 of the children (39%) and both parents of 51 of the children (45%) gave their consent and were assessed, resulting in 261 datasets or an overall response rate of 73%. Only complete triplets of child, mother, and father (*N*=153) were included in this study. Two thirds of the children were female. Children were *m*=14.3 (SD = 2.0, *R*=10–17) years of age, mothers *m*=42.1 (SD = 5.6, *R*=31–59), and fathers *m*=46.6 (SD = 6.0; *R*=35–60) on average. Children had a mean of *m*=8.1 years of education (SD = 1.9, *R*=5–11), mothers *m*=9.0 years (SD = 3.3; *R*=1–20) and fathers *m*=11.7 years (SD = 3.4, *R*=4–19). A total of 59% of fathers compared to 18% of mothers were working at least part-time or undergoing vocational training at the time of the interview. Complete triplets showed no significant difference as compared to incomplete triplets in terms of sex distribution (children), age and symptom scores of PTSD, and depression of children, mothers, and fathers.

### Measures

Socio-demographic sample characteristics were assessed using a brief structured interview. Symptom severity was assessed by means of self-report questionnaires applied by the interviewers by one-to-one verbal administration, in which the instructions and questions were read to the participants. As no validated Albanian versions of the questionnaires were available, all instruments were translated from the original language, and back-translated by native Albanian-speaking interpreters. A native Albanian-speaking psychiatrist compared source and back-translation and adapted the translations where necessary.

#### Children

Traumatic exposure and symptoms of PTSD were established using the UCLA PTSD Reaction Index for DSM-IV, UPID (Pynoos, Rodriguez, Steinberg, Stuber, & Frederick, 1998; Steinberg, Brymer, Decker, & Pynoos, 2004), a widely used screening instrument for children and adolescents. The exposure items are scored as present or absent. The 19 symptom items are scored on a 0–4 Likert scale. A cut-off of 38 showed the greatest sensitivity and specificity for detecting PTSD. Cronbach's alpha has been found to fall in the range of 0.90 (Steinberg et al., 2004) and was 0.86 in our sample.

The Spence Children's Anxiety Scale (SCAS) (Spence, 1998; Spence, Barrett, & Turner, 2003) was developed to assess the severity of anxiety symptoms according to the DSM-IV. The scale assesses six domains of anxiety including generalized anxiety, panic/agoraphobia, social phobia, separation anxiety, obsessive–compulsive disorder, and physical injury fears. The 44 items of the measure are rated on a 4-point Likert scale. Total score and subscale scores are computed by adding the individual item scores. In this study, the subscales of panic/agoraphobia, separation anxiety, and social phobia were used with the recommended cut-offs of six (panic, agoraphobia), six (separation anxiety) and seven (social phobia), respectively (Muris, Schmidt, & Merckelbach, 2000). An anxiety disorder was presumed to be likely if at least one of the three cut-offs was surpassed. Internal consistency was 0.93 for the total scale and 0.74–0.82 for the subscales (Spence, 1998). Cronbach's alpha in our sample was 0.88.

The Depressionsinventar für Kinder und Jugendliche (DIKJ; depression inventory for children and adolescents) (Stiensmeier-Pelster, Schürmann, & Duda, 1989, 2000) is a 26-item questionnaire derived from the Children's Depression Inventory (CDI) (Kovacs, 1985, 1992) and assesses depressive symptoms of children and adolescents aged from 7 to 17 years on a 3-point Likert scale. With a total score ranging from 0 to 52, a cut-off score of ≥12 is recommended to indicate clinically relevant depression (Fruhe, Allgaier, Pietsch, & Schulte-Korne, 2012). Concerning internal consistency, a Cronbach's alpha of 0.81 was found in our sample.

#### Parents

The Posttraumatic Diagnostic Scale (PDS) (Foa, 1995; Foa, Cashman, Jaycox, & Perry, 1997) is a 49-item self-report questionnaire to measure the severity of PTSD symptoms related to a single identified traumatic event. Part I is a trauma checklist that asks about a variety of potentially traumatic events (PTEs) and which in this study was extended by additional traumatic event items from the first part of the Harvard Trauma Questionnaire (Mollica et al., 1992), resulting in a total of 23 items rated as whether experienced, witnessed, or not experienced/witnessed. Part II evaluates the stressor criteria A1 and A2 according to the DSM-IV. Part III assesses the 17 PTSD symptoms experienced in the month prior to assessment. The PDS yields a total severity score ranging from 0 to 51. A probable categorical diagnosis of PTSD can be made with an algorithm following DSM-IV diagnostic criteria for PTSD. Cronbach's alpha in our sample was 0.94 in both mothers and fathers.

The Hopkins Symptom Checklist (HSCL-25) (Mollica et al., 1991) is a well-known and widely used screening instrument, which measures symptoms of anxiety and depression. The shortened 25-item version derived from the 90-item questionnaire (Derogatis, Lipman, Rickels, Uhlenhuth, & Covi, 1974) has been translated into many languages and has been used in different cultural contexts (Mollica, Wyshak, De Marneffe, Khuon, & Lavelle, 1987; Ventevogel et al., 2007). Part I has 10 items addressing anxiety symptoms; Part II has 15 items for depression symptoms. Items are rated on a 4-point Likert scale; the period of reference is the last week. The respondent's total score is the average of all 25 items, while the subscores are the average of the anxiety and depression items, respectively, ranging each from 1.00 to 4.00 (Mollica et al., 1991). The validity of the often-used 1.75 cut-off criterion has been evaluated in relation to different diagnostic psychiatric interviews around the world and found to be accurate (Mollica, Wyshak, De Marneffe, Khuon, & Lavelle, 1987; Veijola et al., 2003). Cronbach's alpha in the present sample was 0.83 (mothers) and 0.93 (fathers) for the anxiety subscale, and 0.90 (mothers) and 0.93 (fathers) for the depression subscale.

### Data analysis

All analyses were conducted using SPSS for Windows, Release 20 (SPSS Inc., Chicago, IL, USA). Descriptive statistics is given in terms of mean and standard deviations in continuous variables, and counts and percentages in categorical variables. Prevalence rates were assessed by counts and percentages of participants exceeding the respective recommended cut-offs. Differences between children and parents were assessed with paired *t*-tests. The relationship between psychopathological and other parameters of children and parents was examined using Pearson's correlations. The regression models (method: enter) included depressive and PTSD symptom scores of children as dependent and parents’ PTSD and depressive symptom scores as independent variables. As anxiety symptoms overlapped with PTSD and showed a lower correlation between children and parents compared to the symptoms of PTSD and depression, they were excluded from the model in order to reduce the number of factors in view of the small sample size. Beta weights, 95% confidence intervals, and adjusted *R* ^2^ are reported. Effect sizes were calculated using Cohen's guidelines (Cohen, 1988). *p*-Values of less than 0.05 were considered significant. Pre-conditions for regression analyses were checked in terms of normal distribution of residuals, autocorrelation of residuals (Durbin-Watson-test), multicollinearity and homoscedasticity and were found to be satisfying for PTSD and depressive symptom scores, respectively.

## Results

### Trauma exposure

The vast majority of children and adults reported having witnessed or experienced one or more PTEs (Table 1). The most frequently reported PTEs in children were *being in a place where war was going on around you* (52.9%), *being beaten up, shot at or threatened to be hurt badly in your town* (35.3%), *hearing about the violent death or serious injury of a loved one* (21.6%), and *seeing someone in your town being beaten up, shot at or killed* (19.6%). The least frequently reported PTEs in children were domestic violence and sexual abuse (2%). Potentially traumatic experiences most frequently reported by fathers and mothers included *lack of shelter* (48%), *lack of food and water* (41.1%), *combat situations* (41.1%), and *being close to death* (33.3%). The least frequently reported PTEs included sexual violence in both fathers and mothers (5%).

**Table 1:** Lifetime trauma exposure (*N*=51 triplets)

	Number of potentially traumatic event types experienced or witnessed										
	
				0		1		2–4		≥5	
							
	*M*	SD	Range	*N*	%	*N*	%	*N*	%	*N*	%
Children	2.39	2.03	0–11	6	11.8	11	21.6	30	58.8	4	7.8
Mothers	4.49	3.29	0–15	4	7.8	7	13.7	17	33.3	23	45.1
Fathers	5.24	4.88	0–21	5	9.8	6	11.8	18	35.3	22	43.1

### Prevalence rates and symptom severity

An overview of prevalence rates is given in Table 2. High rates of posttraumatic stress, anxiety, and depressive symptom scores exceeding the respective cut-offs were found in children and parents. Prevalence rates of children were lower with respect to PTSD and depression but not anxiety as compared to their parents. Mothers suffered more frequently than fathers from clinically relevant anxiety and depression but not from PTSD symptoms. Symptom scores in children were *m*=9.7 (SD = 8.9) for PTSD, *m*=13.1 (SD = 8.3) for anxiety and *m*=8.0 (SD = 6.0) for depression. Symptoms scores in mothers were *m*=11.6 (SD = 13.0) for PTSD and *m*=2.0 (SD = 0.7) for each anxiety and depression. Symptom scores in fathers were *m*=8.7 (SD = 10.2) for PTSD, *m*=1.7 (SD = 0.7) for anxiety and *m*=1.6 (SD = 0.6) for depression. Accordingly, mothers had significantly higher symptom levels of anxiety (*p*=0.004) and depression (*p*<0.001), but not PTSD (*p*=0.136) compared to their husbands. Symptom scores of children and parents were not compared due to the different instruments applied.

**Table 2:** Prevalence of posttraumatic stress, anxiety, and depression (*N*=51 triplets)

	Children		Mothers		Fathers		*p*		
				
Prevalence	*N*	%	*N*	%	*N*	%	C–M	C–F	M–F
PTSD	5	9.8	17	33.3	14	27.5	0.006**	0.028*	0.497
Anxiety	26	51.0	31	60.8	21	41.2	0.322	0.280	0.017*
Depression	10	19.6	29	56.9	19	37.3	0.000***	0.028*	0.024*

* *p*<0.05

** *p*<0.01

*** *p*<0.001.

C–M: children–mother; C–F: children–father; M–F: mother–father.

PTSD: UCLA PTSD Reaction Index for DSM-IV (Children), Posttraumatic Diagnostic Scale (Parents) Anxiety: Spence Children's Anxiety Scale (Children), Hopkins Symptom Checklist-Anxiety (Parents) Depression: Depressionsinventar für Kinder und Jugendliche (Children), Hopkins Symptom Checklist-Depression (Parents).

### Correlation analyses

An overview of correlations between parents’ and children's parameters is given in Table 3. The strongest relationship was found between children's depressive symptoms and fathers’ symptom scores of PTSD, anxiety, and depression. Another significant correlation was found between PTSD symptoms of fathers and children. Maternal symptoms did not correlate with their children's in any respect. Symptom scores of depression, anxiety, and PTSD of fathers correlated significantly with those of mothers. A significant negative correlation was found between paternal education and depressive symptoms of children as well as of mothers. Neither parents’ nor children's trauma exposure correlated with children's symptoms. Boys differed significantly from girls only with respect to anxiety symptoms (slightly higher scores in girls) and trauma exposure (slightly higher in boys).

**Table 3:** Pearson's correlations between parents’ and children's parameters (*N*=51 triplets)

	1	2	3	4	5	6	7	8	9	10	11	12	13	14	15	16	17	18	19
1 C Sex																			
2 C Age	0.027																		
3 C Anx	0.351*	−0.151																	
4 C Depr	−0.171	−0.009	0.445***																
5 C PTSD	−0.004	0.297*	0.438***	0.348*															
6 C Trauma	−0.338*	0.240	−0.167	−0.050	0.169														
7 M Age	0.129	0.337^*^	−0.064	−0.185	−0.040	−0.189													
8 M Empl	0.219	0.075	0.007	0.016	0.001	−0.130	0.151												
9 M Edu	0.205	−0.130	0.019	−0.045	−0.119	0.005	0.013	0.540***											
10 M Anx	0.125	−0.099	−0.055	−0.001	0.124	0.042	−0.174	−0.161	−0.051										
11 M Depr	0.018	−0.032	0.035	0.226	0.147	0.067	−0.148	−0.147	−0.171	0.757***									
12 M PTSD	0.193	0.047	0.140	0.090	0.053	−0.117	−0.138	−0.091	−0.259	0.408**	0.627***								
13 M Trauma	0.004	0.177	0.037	0.104	0.259	0.282*	0.124	0.178	−0.035	0.316*	0.324*	0.174							
14 F Age	0.059	0.256	−0.061	−0.140	0.006	−0.208	0.797***	0.251	0.059	−0.057	−0.106	−0.061	0.157						
15 F Empl	0.054	−0.025	0.093	0.129	−0.208	0.047	−0.119	0.213	0.223	−0.232	0.010	−0.037	0.107	−0.023					
16 F Edu	0.067	0.101	−0.183	−0.466***	−0.204	0.196	0.263	0.063	0.360**	−0.216	−0.307*	−0.164	−0.138	0.214	0.250				
17 F Anx	−0.142	−0.183	0.102	0.434***	0.234	−0.095	−0.112	−0.161	−0.146	0.484***	0.518***	0.254	0.161	0.062	−0.089	−0.390**			
18 F Depr	−0.149	−0.260	0.209	0.441***	0.244	−0.096	−0.094	−0.240	−0.250	0.417**	0.627***	0.371**	0.184	−0.028	−0.047	−0.470***	0.836***		
19 F PTSD	−0.242	−0.026	0.217	0.509***	0.299*	0.053	−0.130	0.052	−0.127	0.274	0.349*	0.335*	0.278*	0.083	−0.133	−0.429**	0.567***	0.535***	
20 F Trauma	−0.120	−0.164	−0.106	0.133	0.178	−0.036	−0.104	−0.140	−0.089	0.378**	0.308*	0.194	0.450***	0.130	0.071	−0.079	0.509***	0.459***	0.308*

* *p*<0.05

** *p*<0.01

*** *p*<0.001

C: Children; M: Mothers; F: Fathers; Empl: Employment; Edu: Education (Years); Anx: Anxiety Total Score (Children: SCAS; Parents: HSCL-25); Depr: Depression Total Score (Children: DIKJ; Parents: HSCL-25); PTSD: PTSD Total Scores (Children: UPID; Parents: PDS); Trauma: Number of Trauma Categories Experienced or Witnessed (lifetime).

### Regression analysis

Results of the regression analysis for PTSD and depressive symptoms in children, mothers, and fathers, controlled for trauma exposure and education, are shown in Table 4. Children's PTSD values were neither related to parental PTSD values nor to their depressive symptoms. Children's depressive symptoms were significantly related to their fathers’ PTSD symptoms but not to their mothers’. Parental depressive symptoms had no significant beta weight for children's depressive symptoms.

**Table 4:** Regression analysis for posttraumatic stress and depressive symptoms in children (*N*=51 triplets)

	Dependent			
	
	Children PTSD		Children depression	
		
Independent	Beta (95%CI)	B (SE)	Beta (95%CI)	B (SE)
Children				
Trauma exposure	0.18 (−0.12; 0.49)	0.80 (0.68)	0.00 (−0.26; 0.26)	0.01 (0.39)
				
Mothers				
PTSD	−0.06 (−0.45; 0.34)	−0.04 (0.13)	−0.09 (−0.43; 0.24)	−0.04 (0.08)
Depression	−0.02 (−0.47; 0.43)	−0.24 (2.88)	−0.04 (−0.42; 0.35)	−0.30 (1.66)
Education	−0.05 (−0.36; 0.27)	−0.12 (0.43)	0.13 (−0.14; 0.39)	0.23 (0.24)
				
Fathers				
PTSD	0.20 (−0.16; 0.56)	0.17 (0.16)	0.33* (0.02; 0.63)	0.19 (0.09)
Depression	0.14 (−0.29; 0.56)	2.02 (3.21)	0.22 (−0.14; 0.58)	2.18 (1.85)
Education	−0.09 (−0.45; 0.27)	−0.24 (0.48)	−0.30 (−0.60; 0.01)	−0.53 (0.28)
	Adjusted *R* ^*2*^=0.002		Adjusted *R* ^*2*^=0.277	
	*F*(7, 43) = 1.01		*F*(7, 43) = 3.73	
	*p*=0.435		*p*=0.003	
	Effect size = 0.03		Effect size = 0.35	

* *p*<0.05

** *p*<0.01

*** *p*<0.001.

## Discussion

This cross-sectional study examined lifetime trauma exposure, trauma-related mental health problems, and their intergenerational relationship in a randomly selected sample of Kosovar school-aged children and their parents (*N*=51 triplets) 11 years after the end of war. High trauma exposure and high prevalence rates of clinically relevant symptoms of PTSD, anxiety, and depression were found in both generations. Children's depressive symptoms correlated with their fathers’ symptoms of PTSD, anxiety, and depression, whereas maternal symptoms did not correlate with their children's. Regression analysis showed a significant relationship between children's depressive and fathers’ posttraumatic stress symptoms.

This study has several limitations. As no structured clinical interviews were performed, the extent to which self-reported symptoms of PTSD, anxiety, and depression would match clinical diagnoses is unclear. Neither validation interviews nor double-ratings of the interviews were conducted, basically because of the self-rating character of the questionnaires. The implementation of invalidated Albanian versions of measurement instruments is another limitation. However, translations and back-translations were thoroughly performed by experienced, native Albanian-speaking interpreters and adapted in a meticulous reconciliation process, if necessary, in order to achieve maximum quality. Cronbach's alphas in our sample were similar to those of the original versions, suggesting comparable reliability of the translated versions. Although girls were overrepresented, we only found a few minor gender differences and we therefore do not expect a relevant bias. As all efforts for optimal randomization were made on regional, communal, school, and participant level and as our findings correspond well with large epidemiological surveys in similar samples, we assume our results can be regarded as fairly representative for Kosovar families with school-aged children in general. The sample size is sufficient for calculating approximate prevalence rates and correlations; however, as the sample was divided into three subgroups, it was too small for extensive multivariate regression analyses. A specific strength of our study is the examination of triplets (children, mother, and father) to investigate intergenerational aspects. Another strength comprises the administration of self-rating instruments by master-level students of psychology, who were not involved in data processing.

As expected, lifetime trauma exposure in adults and children was high compared to non-conflict countries (Kessler, Sonnega, Bromet, Hughes, & Nelson, 1995a; National Institute of Mental Health, 2012; Perkonigg, Kessler, Storz, & Wittchen, 2000): only 10% of children and parents reported no traumatic experiences whereas barely 10% of children and almost half of parents reported having experienced or witnessed five or more PTE types. More than half of the children reported war-related experiences. The high prevalence rates of clinically relevant symptoms of PTSD (10, 33, and 27%, respectively), anxiety (51, 61, and 41%, respectively),and depression (20, 57, and 37%, respectively) in children, mothers, and fathers of our sample are in line with earlier studies conducted in the postwar Balkans (Kashdan et al., 2009; Morina & Ford, 2008; Priebe et al., 2010). Notably, the long time lapse of more than a decade since the end of the war seems not to result in a considerable convergence to non-conflict civilian populations with respect to mental health problems. One of the reasons might be the limited availability of mental healthcare resources (Jones, Rrustemi, Shahini, & Uka, 2003). Providing specific and culturally appropriate screening and treatment programs, in turn, would be crucial in order to prevent further long-term impairment and secondary harm. A school-based mental health approach, as performed in this study, turned out to be feasible, both logistically and economically, for assessments on community and population levels.

With regard to the intergenerational interplay of trauma exposure and PTSD, parental trauma exposure was not related to their children's PTSD symptoms, whereas paternal (but not maternal) PTSD symptoms correlated significantly with children's PTSD symptoms. Literature in this regard is inconsistent. The comparison of 50 children of male Vietnam veterans with and without PTSD with an age-matched group of 33 civilian peers found no significant difference in terms of PTSD symptomatology for any offspring group, whereas the effect of veteran's PTSD appeared to manifest itself particularly in the area of unhealthy family functioning (Davidson & Mellor, 2001). A review of 35 comparative studies on the mental state of offspring of Holocaust survivors indicated that the non-clinical population of children of Holocaust survivors did not show more psychopathological symptoms than people in general while the clinical population of offspring tended to present a specific profile including a predisposition to PTSD (Kellerman, 2001). Yehuda et al., in turn, found that adult offspring of Holocaust survivors had a greater prevalence of current and lifetime PTSD and other psychiatric diagnoses than demographically similar comparison subjects (2001) and demonstrated a specific association between parental PTSD and the occurrence of PTSD in offspring (1998). The association of paternal PTSD symptoms and depressive symptoms of children seen in our sample is in line with previous studies suggesting parental trauma exposure and PTSD being unspecific mediating rather than causal factors, that potentially, but not necessarily, contribute to a range of psychiatric problems in offspring including other axis I disorders, behavioral and developmental abnormalities (Dekel & Goldblatt, 2008; Galovski & Lyons, 2004; Yehuda, Bell, Bierer, & Schmeidler, 2008; Yehuda et al., 2001).

Our hypothesis of equal correlations of maternal and paternal mental health with that of their children could not be supported. In spite of the higher symptom load compared to fathers, maternal mental health did not correlate at all with their children's, whereas a significant relationship was found between paternal symptoms of PTSD, anxiety, and depression and their children's depressive symptoms, as well as between PTSD symptoms of fathers and children. Furthermore, in regression analysis, paternal but not maternal PTSD symptoms were significantly related to children's depression (adjusted *R* ^2^ =0.277). These findings have, to our knowledge, not been reported in the literature to date. Yehuda et al. (2008) in contrast found that maternal, not paternal, PTSD was related to an increased risk for PTSD in 211 adult offspring of Holocaust survivors, while PTSD in any parent contributed to the risk for depression, and parental traumatization was associated with increased anxiety disorders. These partially contradictory findings are not surprising given the heterogeneity of the investigated samples, the variety of potential resilience and risk factors and the many conceivable pathways of intergenerational interplay, ranging from genetic and epigenetic to psychological, social, and cultural mechanisms. The findings in our Kosovar sample may possibly be explained by a specifically momentous position of fathers within the family structure: a large review recently found that perceived paternal rejection, as could be expected in case of fathers with mental disorders, is associated with negative personality development, that is, in terms of self-esteem and emotional stability (Khaleque & Rohner, 2012). The hypothesis is supported by the correlations of fathers’ symptoms of depression, anxiety, and PTSD with those of their wives and the negative correlation of paternal education with depression of mothers and children, indicating higher paternal education being a protective factor. A complementary hypothesis, though speculative and not directly supported by the data of this study, could imply different ways of expression of mental health problems, particularly PTSD, among parents: as compared to mothers, fathers might present with rather externalizing behavior (Miller, Greif, & Smith, 2003; Rielage, Hoyt, & Renshaw, 2010), resulting in a higher impact on family members. The higher scores of maternal symptoms of anxiety and depression as indicators of an internalizing personality style would support this assumption. Future research should test the hypothesis given its potential for relevant clinical implications.

### Clinical implications and conclusions

More than a decade after the end of the Kosovo war, the presence of posttraumatic stress, anxiety, and depressive symptoms in civilian adults and their children is still substantial, corresponding to a high lifetime trauma exposure. Symptoms of parents (here: fathers) and children are associated. Therefore, mental health problems of close ones should actively be screened and accounted for in comprehensive healthcare plans, using a systemic approach. This seems particularly important when mental health programs are addressing selective risk groups. The intergenerational interplay of trauma-related mental health problems is a complex, non-linear process and is not sufficiently defined by the severity and distribution of mental disorders within families. Future research should include longitudinal studies conducting multivariate analyses with larger sample sizes in order to investigate indicators, causal and resilience factors.
